# Seasonal variation in host susceptibility and cycles of certain infectious diseases.

**DOI:** 10.3201/eid0703.010301

**Published:** 2001

**Authors:** S F Dowell

**Affiliations:** Centers for Disease Control and Prevention, Atlanta, Georgia 30333, USA. sdowell@cdc.gov

## Abstract

Seasonal cycles of infectious diseases have been variously attributed to changes in atmospheric conditions, the prevalence or virulence of the pathogen, or the behavior of the host. Some observations about seasonality are difficult to reconcile with these explanations. These include the simultaneous appearance of outbreaks across widespread geographic regions of the same latitude; the detection of pathogens in the off-season without epidemic spread; and the consistency of seasonal changes, despite wide variations in weather and human behavior. In contrast, an increase in susceptibility of the host population, perhaps linked to the annual light/dark cycle and mediated by the pattern of melatonin secretion, might account for many heretofore unexplained features of infectious disease seasonality. Ample evidence indicates that photoperiod-driven physiologic changes are typical in mammalian species, including some in humans. If such physiologic changes underlie human resistance to infectious diseases for large portions of the year and the changes can be identified and modified, the therapeutic and preventive implications may be considerable.


### Seasonal Variation in Host Susceptibility and Cycles of Certain Infectious Diseases

**Scott F. Dowell**
Centers for Disease Control and Prevention, Atlanta, Georgia, USA

Seasonal cycles of infectious diseases have been variously attributed to changes in atmospheric conditions, the prevalence or virulence of the pathogen, or the behavior of the host. Some observations about seasonality are difficult to reconcile with these explanations. These include the simultaneous appearance of outbreaks across widespread geographic regions of the same latitude; the detection of pathogens in the off-season without epidemic spread; and the consistency of seasonal changes, despite wide variations in weather and human behavior. In contrast, an increase in susceptibility of the host population, perhaps linked to the annual light/dark cycle and mediated by the pattern of melatonin secretion, might account for many heretofore unexplained features of infectious disease seasonality. Ample evidence indicates that photoperiod-driven physiologic changes are typical in mammalian species, including some in humans. If such physiologic changes underlie human resistance to infectious diseases for large portions of the year and the changes can be identified and modified, the therapeutic and preventive implications may be considerable.

From 1703 onward, the annual rise and fall of measles deaths in London was recorded in sufficient detail to allow for careful mathematical modeling in 1918 (1). Since then, surveillance for a variety of diseases has established that regular seasonal variation in incidence is the rule, rather than the exception, for acute infections. Seasonal variations should be distinguished from periodic large epidemics, as observed every 2 years for measles (2) or at less frequent and more irregular intervals for meningococcal meningitis (3) and rubella (4). This discussion will focus on the more robust annual cycle, which "locks in" large epidemics to the same time of year (3,4) and persists even after large epidemics have been eliminated by mass vaccination (2). The life cycles of pathogens spread by insect vectors or maintained in animal or environmental reservoirs add complexity because seasonal changes might influence not only the pathogen or human host but also the vector population and animal or environmental reservoir. Therefore, this discussion will focus on bacterial and viral pathogens maintained primarily by person-to-person spread.

The regular and predictable pattern of seasonal outbreaks dominates the epidemiology of many exclusively human pathogens (Figure 1). Different infections peak in each of the four seasons, but for each pathogen, the timing and characteristics of the annual outbreak are remarkably consistent from year to year. Other key observations have been made on the seasonality of infectious diseases, including the simultaneous onset of outbreaks in geographically remote areas and the persistence of pathogens in the off-season in the absence of epidemic spread (Table). In fact, latitude has a clear influence on the timing and magnitude of outbreaks of rotavirus infection (10), influenza (15), and poliomyelitis

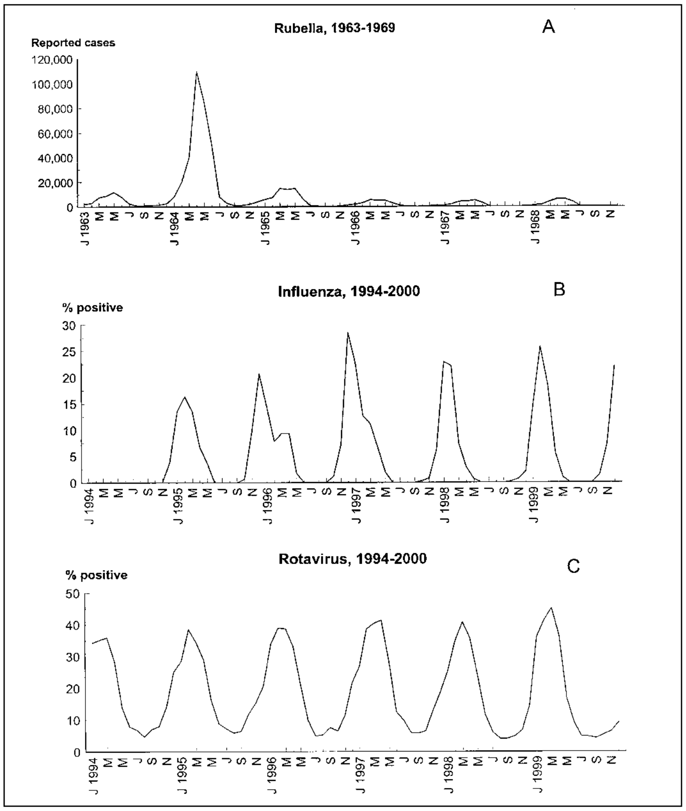

Figure 1. Seasonal variation in the occurrence of three human pathogens in the U.S. A: an annual cycle of rubella activity was maintained between larger epidemics, which occurred every 6 to 9 years. B: percentage of specimens testing positive for influenza viruses among specimens tested by World Health Organization and U.S. National Respiratory and Enteric Virus Surveillance System collaborating laboratories. C: a consistent pattern of rotavirus seasonality is evident in the U.S. National Respiratory and Enteric Virus Surveillance System. Adapted from references 4-6.

Address for correspondence: Scott F. Dowell, Centers for Disease Control and Prevention, 1600 Clifton Road NE, Mailstop C12, Atlanta, GA 30333, USA; fax: 404-639-3039; e-mail: sdowell@cdc.gov

Table. Observations on the seasonal occurrence of infectious diseases

| Observation | Examples |
|---|---|
| Pathogens peak at characteristic times in all seasons of the year | Winter: influenza, pneumococcus, rotavirus<br>Spring: RSV, measles<br>Summer: polio, other enteroviruses<br>Fall: parainfluenza virus type 1 |
| Timing and duration of peaks for each pathogen are similar from year to year | Measles: regular pattern since 1703 (1)<br>Influenza: annual peak varies by only 5 to 10 weeks in the United States (6) |
| Onset of epidemics often occurs simultaneously in areas that are geographically dispersed and have different weather conditions and diverse populations | Influenza: simultaneous outbreaks across North America, 16 European countries, and 6 Chinese provinces (7)<br>Pneumococcus: simultaneous outbreaks in seven surveillance areas (8) |
| Latitude is a critical determinant of timing and magnitude of peaks | An increasing magnitude of seasonal peaks as distance from the equator increases has been documented for polio (9) and rotavirus (10) and reported for influenza (11). |
| Pathogens can be detected in the off-season despite lower incidence of disease and virtual absence of epidemics | Meningococcus: no decrease in carriage in the off-season, despite absence of epidemic disease (12)<br>RSV: sporadic summer viral isolation but no epidemic spread (13)<br>Influenza: sporadic summer isolation, occasional clusters of disease without epidemic spread (14) |

RSV = respiratory syncytial virus. RSV peaks in the winter or spring in the United States, depending on location. For simplicity, it is listed here as a spring pathogen.

(Figure 2) (9). Reconciling these observations with the consistent seasonality of clinical illness is a continuing challenge.

### Explanations of Seasonality

Because seasonal cycles of infectious diseases are so universal and no single theory has proved satisfactory, explanations about their cause abound. More than one explanation or combination of explanations may be true. Explanations can be grouped into three types: pathogen appearance and disappearance, environmental changes, and host-behavior changes.

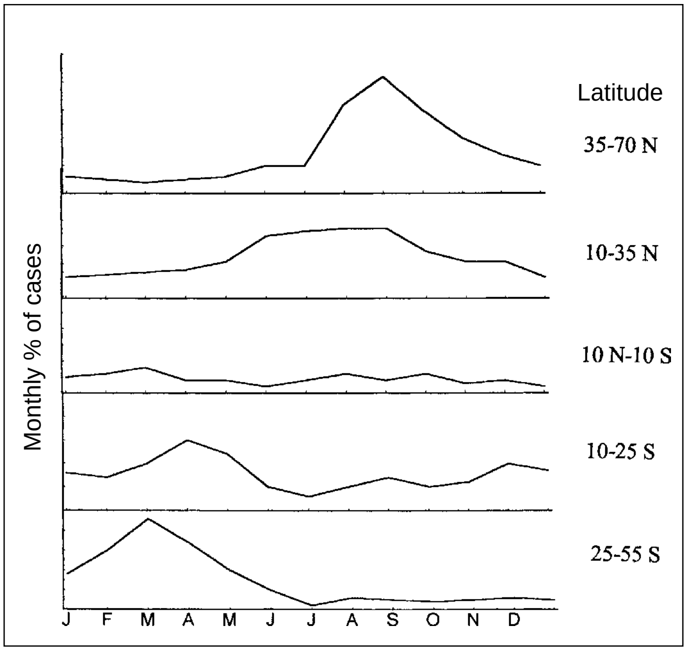

Figure 2. Seasonal variation in the incidence of poliomyelitis by latitude, 1956-57. As distance from the equator increases, a higher proportion of cases are evident in summer and fall months. Adapted from reference 9.

### Pathogen Appearance and Disappearance

Perhaps the most obvious explanation for the absence of disease during a period is that the pathogen is also absent during the period. However, the regular annual migration of epidemics of influenza, poliomyelitis, and rotavirus infection from northern latitudes across the equator to southern ones and back does not necessarily imply that the pathogens themselves migrate in this way.

Current theory holds that influenza is maintained only by direct spread in a series of chains of transmission from one ill person to another (16). Some evidence suggests that influenza viruses do spread geographically, particularly during pandemics, but whether geographic spread accounts for the patterns observed in annual outbreaks has been questioned (11,17,18). The simultaneous onset of geographically widespread outbreaks is difficult to reconcile with chains of person-to-person transmission. One hypothesis is that earlier "seeding" of the virus throughout the population must have occurred (17). During an 1826 influenza epidemic, one observer wrote, "...this epidemic affects a whole region in the space of a week, nay, a whole continent as large as North America, together with all the West Indies, in the course of a few weeks, while the inhabitants could not within so short a time have had any communication or intercourse whatever across such a vast extent of country" (11). A more recent hypothesis attributes geographic spread to the atmospheric dispersion of virus from Southeast Asia by trans-Pacific winds across the North American continent (18).

### Environmental Changes

Environmental changes, particularly changes in weather, are the explanations most often invoked for the seasonality of infectious diseases. Statistically significant correlations between epidemic cycles and cycles of temperature (19-22), humidity (21-23), rains (24), or winds (24) have been identified. However, correlations may be found with confounders as well as with causes.

In some cases, the association with weather is supported, but the biologic plausibility appears tenuous. Although the seasonal incidence of poliomyelitis correlated quite well with the summer increase in relative humidity in Boston and Houston from 1942 to 1951 (23), the explanation that aerosolized poliovirus survives for a longer time at higher relative humidity is difficult to reconcile with the fecal-oral route of poliovirus transmission.

In other cases, the correlations are supported by biologic plausibility but are not consistently observed. In sub-Saharan Africa, the onset of meningococcal epidemics closely followed the season of dry winds and ended with the onset of the rains (25). It has been proposed that drying of mucosal surfaces increases the probability of bacteremic spread and that the rains moisten the mucosa or decrease the spread of the organism by dust. However, in Oregon and other areas, meningococcal disease peaks during the rainy season (26). Similarly, a significant correlation between the onset of the invasive pneumococcal disease season and a drop in mean daily temperatures below 24˚C in Houston (19) was not confirmed in seven other areas with more widely varying weather patterns (8). Respiratory syncytial virus epidemics occur in the colder months of winter and spring in the United States (13) but paradoxically are significantly correlated with the hotter months in Singapore and Hong Kong (21,22).

### Host-Behavior Changes

Seasonal changes in poliomyelitis, measles, and other seasonal infectious diseases have been attributed to changes in the behavior of the host. Public swimming pools were a source of great concern during the polio epidemics of the 1950s, and summer peaks in polio and other enteroviruses were attributed to swimming (23,27,28). Subsequent studies discounted the importance of swimming in the spread of enterovirus infections (28).

Crowding of susceptible persons is one of the most common explanations for seasonal infectious diseases, and it certainly has biologic plausibility. The seasonal patterns of measles in England and Wales have been attributed to the timing of school holidays (29,30). Although such explanations are plausible, one must also ask why influenza outbreaks do not occur in crowded international conventions during summer, and why measles outbreaks are not common at summer camps. As one authority noted regarding meningococcal seasonality, "The story that African epidemics are caused by people crowding together at night during the dry season is a medical myth which is difficult to kill. Villagers sleep inside at the height of the rainy season at least as frequently as during the cold part of the dry season..." (24).

Comprehensive explanations of seasonal changes in infectious diseases should identify the means by which similar pathogens peak at different seasons (with characteristic timing and duration) and explain the prompt regionwide epidemics in geographically dispersed populations, the variation in epidemic patterns by latitude, and the persistence of the pathogen in the off-season without epidemic disease (Table).

### The Proposed Hypothesis

Regular annual variations in the incidence of many infectious diseases may be due to changes in susceptibility of the human host to the particular pathogen. Like the seasonal physiologic cycles of many mammalian species, these changes in susceptibility may be timed to the light/dark cycle, typically mediated by changes in the duration of the daily melatonin pulse. The changes in susceptibility may be distinct for different pathogens and may cover a broad range of possibilities, including (but not limited to) changes in the characteristic of mucosal surfaces, the expression of epithelial receptors, the leukocyte numbers or responsiveness, or other features of specific or nonspecific immunity.

This hypothesis would predict that pathogens do not physically migrate across the equator and that nationwide epidemics do not necessarily result from chains of person-to-person transmission. Rather, the pathogens may be present in the population year-round, and epidemics occur when the susceptibility of the population increases enough to sustain them. Perhaps the most significant prediction is that people are relatively resistant to disease if exposed in the off-season and that the specific physiologic process leading to seasonal resistance should be identifiable and perhaps modifiable.

### Seasonal Changes in Host Physiology

Many mammalian species undergo seasonal physiologic changes. The best characterized are changes in reproductive organs and other tissues seen in animals that are seasonal breeders. Humans are not seasonal breeders, but fertility has seasonal variations. Seasonal variations have been documented in other physiologic processes and immunologic features (31,32).

Producing offspring in a season during which food is unavailable and the environment is unsuitable for the young is an evolutionary dead-end for some species, leading to carefully regulated breeding seasons for many rodents (33), sheep (34), other ungulates (35), monkeys (36), and primates (37). Seasonal physiologic changes involve not just behavior but also the secretion of sex hormones and the size and function of reproductive organs. In controlled laboratory conditions, the duration of the light/dark cycle is the key parameter governing these seasonal changes, which can be completely replicated by artificial manipulation of the photoperiod. Photoperiod is most commonly used rather than temperature, humidity, food availability, or other seasonally varying parameters, presumably because its invariant nature best prevents accidental breeding at the wrong time of year. Under constant photoperiod, the physiologic changes can also be reproduced by controlling the duration of the daily melatonin pulse.

Seasonal physiologic changes have also been documented in processes not typically associated with breeding but potentially related to susceptibility to infectious agents. For example, even under constant conditions, red deer have distinct seasonal changes in digestive features (35), mice have seasonal changes in seizure threshold (38), and dairy cattle have seasonal changes in the fat and protein content of their milk (39). In recent years, seasonal changes in immunologic features have been documented. For example, Siberian hamsters exposed to short-day photoperiod demonstrate increased natural killer-cell activity and lymphocyte blastogenesis but decreased phagocytosis and oxidative burst activity by granulocytes (40); deer mice treated with melatonin in constant photoperiod exhibit increased lymphocyte response to mitogen stimulation (41).

A series of studies documented that the death rate in mice experimentally exposed to pneumococcal infection varied with the time of day (42-44). Survival patterns were altered by modifying environmental lighting conditions, rather than feeding or activity, and susceptibility appeared related to the daily cycle of cortisone, although the specific physiologic feature responsible for increased susceptibility was not identified. Since these findings, understanding of the role of melatonin and its control of circadean and seasonal rhythms has increased greatly, but further studies of the influence of photoperiod on experimental pneumococcal infections in mice appear not to have been pursued.

Seasonal physiologic changes are not as well characterized for humans as for other mammals, but mounting data suggest that changes in photoperiod and the melatonin pulse may also influence human physiology (32). Blind people, who lack the capability for light to cue their biologic clocks, are often plagued by free-running circadian rhythms. A recent study demonstrated that these free-running rhythms can be entrained to a normal cycle by daily administration of melatonin (45). Although humans are sexually active year-round, a seasonal distribution in conceptions has consistently been demonstrated, and a variation in the ovulation rate has been postulated as the cause (31). Seasonal affective disorder, a well-characterized depression associated with short days and specific genetic defects (46), is treatable with extra hours of exposure to broad-spectrum light (47). Seasonal variations in heart attacks (48), breast cancer (49), and other seemingly noninfectious conditions have also been reported.

Recent research has focused on seasonal changes in immunologic values in humans. Specific melatonin receptors coupled with G-protein have been identified on lymphocytes (50). As in rodents, seasonal variations in lymphocyte mitogenic responses and in the quantity of circulating lymphocytes, neutrophils, CD4 and CD8 cells, and IL-6 have been reported (51-53). Some values, such as lymphocyte aryl hydrocarbon hydroxylase activity, peak in summer (54), while others, such as number of circulating B cells, peak in winter (52). Although statistically significant, the functional significance of these variations has not yet been established.

### Testing the Hypothesis

The above observations lend some biologic plausibility to the proposed hypothesis, but direct testing is needed. Several observations support the prediction that the host is less susceptible to infection or disease in the off-season.

In a double-blind placebo-controlled trial conducted in the Soviet Union during different seasons, nonimmune volunteers were given attenuated live influenza vaccine intranasally (55). Febrile reactions attributable to vaccine (calculated by subtracting the proportion of participants with reactions in the placebo group from the proportion in the vaccine group) were observed in 6.7% of 360 volunteers inoculated in Leningrad in January, compared with 0.8% of 197 inoculated in June ($p = 0.003$). Fourfold rises in antibody titer were seen in 31% to 40% in Krasnodar in January, depending on the vaccine strain, compared with 4.3% to 4.8% given the same strains in May and October (all $p < 0.001$). Similar trends with less significant differences were seen in three other cities.

Some years earlier, in a series of experiments on the transmission of influenza virus from infected to susceptible mice, <1% of mice exposed from July to October were infected, compared with 22% of those exposed in December or January ($p < 0.001$) (56). One year later, the investigators repeated the experiment with a different strain of mice, now kept under constant temperature and humidity, and observed that 34% were infected in May to October, compared with 58% in November to April ($p < 0.001$). The photoperiod conditions in these experiments were not noted.

It is not clear whether attempts were made to replicate these provocative experiments or if the potential importance of the observations was fully appreciated. The animal experiments may be relatively easy to confirm or refute, and the many live attenuated vaccines currently tested or used should provide ample material to evaluate the effects of season on immunogenicity or reactogenicity. The season of administration influences seroconversion rates to oral polio vaccine (57,58) and protection against polio (59), but much of this seasonal variation may be attributable to competition by other enteroviruses during summer (57). Vaccine-associated paralytic polio among vaccine contacts reflects the seasonal pattern of natural polio (60).

### Conclusion

Photoperiod-driven changes in host physiology might explain certain enigmatic observations about seasonality, but some observations remain unexplained. For example, the west-east movement of rotavirus is not easily attributable to host susceptibility changes timed to the light/dark cycle (5). The increase in hospitalizations coincident with warm weather and El Nino points to temperature rather than photoperiod as a key influence on some diarrheal disease pathogens (20). The sudden appearance and worldwide spread of a new pandemic strain of influenza virus also argues more for chains of transmission than for a crop of outbreaks from virus already present in the population.

Epidemiologists have long puzzled over why seasonal infectious disease outbreaks occur when they do. Perhaps the more important question is why they do not occur when they do not. Is the human population already relatively resistant for 6 to 9 months each year? If the absence of epidemics of summer influenza or winter polio is attributable to climate or weather, we may have little power to influence them. On the other hand, if these annual troughs are due to increased host resistance, opportunities abound for studying and modifying these changes. Such opportunities might include reviews of existing databases, careful evaluation of “experiments of nature,” and studies in laboratory animals.

Databases surely exist that might shed light on this hypothesis. Clinical trials of live attenuated vaccines during the usual seasonal peak and seasonal trough for that particular disease could be reviewed for seasonal differences in reactogenicity and immunogenicity. Experiments of nature, in which groups adapted to summer come into contact with groups adapted to winter (as in a convention or a cruise ship with passengers from both Southern Hemisphere and Northern Hemisphere countries) and are exposed to a seasonal pathogen (such as influenza or an enterovirus), could be analyzed for differences in attack rate or clinical severity. Laboratory animals housed in photoperiod-controlled rooms could be exposed to seasonal pathogens and evaluated to see if photoperiod or melatonin modifies clinical and physiologic responses to infection. If differences are documented, the specific physiologic feature governing susceptibility changes could be isolated and identified.

It is time to have a closer look at these possible seasonal changes in host susceptibility and if they are confirmed, identify and modify the physiologic changes underlying annual cycles of infectious diseases.

### Acknowledgments

The author thanks Anne Schuchat, G. Robert Lynch, Marc Fischer, Stephanie Schrag, Alicia Fry, and David Shay for helpful discussions and suggestions on the manuscript.

Dr. Dowell is acting Associate Director for Global Health, National Center for Infectious Diseases, CDC. His research focuses on the epidemiology of infectious diseases.

### References

1. Brownlee J. An investigation into the periodicity of measles epidemics in London from 1703 to the present day by the method of the periodogram. Philosophical Transactions of the Royal Society of London 1918;B 208:225-50.
2. Anderson R, Grenfell BT, May RM. Oscillatory fluctuations in the incidence of infectious disease and the impact of vaccination: time series analysis. J Hyg Camb 1984;93:587-608.
3. Riedo F, Plikaytis B, Broome C. Epidemiology and prevention of meningococcal disease. Pediatr Infect Dis J 1995;14:643-57.
4. Witte J, Karchmer A, Case M, Herrmann KL, Abrutyn E, Kassanoff I, et al. Epidemiology of rubella. Am J Dis Child 1969;118:107-11.
5. Torok TJ, Kilgore PE, Clarke MJ, Holman RC, Bresee JS, Glass RI. Visualizing geographic and temporal trends in rotavirus activity in the United States, 1991 to 1996. National Respiratory and Enteric Virus Surveillance System Collaborating Laboratories. Pediatr Infect Dis J 1997;16:941-6.
6. Centers for Disease Control and Prevention. Update: Influenza activity—United States, 1999-2000 season. MMWR Morb Mortal Wkly Rep 2000;49:173-7.
7. Centers for Disease Control and Prevention. Update: Influenza activity—United States and worldwide, 1995-96 season, and composition of the 1996-97 influenza vaccine. MMWR Morb Mortal Wkly Rep 1996;45:326-9.
8. Dowell S, Whitney C, Wright C, Schuchat A. Seasonal changes in invasive pneumococcal disease. Emerg Infect Dis. In press 2001.
9. Paccaud M. World trends in poliomyelitis morbidity and mortality, 1951-1975. World Health Stat Quart 1979;32:198-224.
10. Cook S, Glass R, LeBaron C, Ho M-S. Global seasonality of rotavirus infections. Bull World Health Organ 1990;68:171-7.
11. Hope-Simpson R, Golubev D. A new concept of the epidemic process of influenza A virus. Epidemiol Infect 1987;99:5-54.
12. Blakebrough I, Greenwood B, Whittle H, Bradley A, Gilles H. The epidemiology of infections due to *Neisseria meningitidis* and *Neisseria lactamica* in a northern Nigerian community. J Infect Dis 1982;146:626-37.
13. Centers for Disease Control and Prevention. Update: respiratory syncytial virus activity—United States, 1998-1999 Season. MMWR Morb Mortal Wkly Rep 1999;48:1104-15.
14. Kohn M, Farley T, Sundin D, Tapia R, McFarland L, Arden N. Three summertime outbreaks of influenza type A. J Infect Dis 1995;172:246-9.
15. Cox NJ, Fukuda K. Influenza. Infect Dis Clin N Am 1998;12:27-38.
16. Cox N, Subbarao K. Influenza. Lancet 1999;354:1277-82.
17. Langmuir A, Schoenbaum S. The epidemiology of influenza. Hosp Pract 1976;11:49-56.
18. Hammond G, Raddatz R, Gelskey D. Impact of atmospheric dispersion and transport of viral aerosols on the epidemiology of influenza. Rev Infect Dis 1989;11:494-7.
19. Kim P, Musher D, Glezen W, Rodriguez-Barradas M, Nahm W, Wright C. Association of invasive pneumococcal disease with season, atmospheric conditions, air pollution, and the isolation of respiratory viruses. Clin Infect Dis 1996;22:100-6.
20. Checkley W, Epstein L, Gilman R, Figueroa D, Cama RI, Patz JA. Effects of El Niño and ambient temperature on hospital admissions for diarrheal diseases in Peruvian children. Lancet 2000;355:442-50.
21. Chew F, Doraisingham S, Ling A, Kumarasinghe G, Lee B. Seasonal trends of viral respiratory tract infections in the tropics. Epidemiol Infect 1998;121:121-8.
22. Sung R, Murray H, Chan R, Davies D, French G. Seasonal patterns of respiratory syncytial virus infection in Hong Kong: a preliminary report. J Infect Dis 1987;156:527-8.
23. Nathanson N, Martin J. The epidemiology of poliomyelitis: enigmas surrounding its appearance, epidemicity, and disappearance. Am J Epidemiol 1979;110:672-92.
24. Greenwood B. The epidemiology of acute bacterial meningitis in tropical Africa. Bacterial Meningitis. London: Academic Press; 1987. p. 61-91.
25. Greenwood B, Blakebrough I, Bradley A, Wali S, Whittle H. Meningococcal disease and season in sub-Saharan Africa. Lancet 1984;i:1339-42.
26. Diermayer M, Hedberg K, Hoesly F, Fischer M, Perkins B, Reeves M, et al. Epidemic serogroup B meningococcal disease in Oregon: the evolving epidemiology of the ET-5 strain. JAMA 1999;281:1493-7.
27. D'Alessio D, Minor T, Allen C, Tsiatis A, Nelson D. A study of the proportions of swimmers among well controls and children with enterovirus-like illness shedding or not shedding an enterovirus. Am J Epidemiol 1981;113:533-41.
28. Hawley H, Morin D, Geraghty M, Tomkow J, Phillips C. Coxsackievirus B epidemic at a boys' summer camp: isolation of virus from swimming water. JAMA 1973;226:33-6.
29. Hamer W. Epidemic disease in England—the evidence of variability and persistency of type. Lancet 1906;11:733-9.
30. Fine P, Clarkson J. Measles in England and Wales - I: an analysis of factors underlying seasonal patterns. Int J Epidemiol 1982;11:5-14.
31. Rojansky N, Brzezinski A, Schenker J. Seasonality in human reproduction: an update. Hum Reprod 1992;7:735-45.
32. Wehr T, Moul D, Barbato G, Giesen HA, Seidel JA, Barker C, et al. Conservation of photoperiod-responsive mechanisms in humans. Am J Physiol 1993;265:R846-57.
33. Carlson L, Zimmermann A, Lynch G. Geographic differences for delay of sexual maturation in *Peromyscus leukopus*: effects of photoperiod, pinealectomy, and melatonin. Biology of Reproduction 1989;41:1004-13.
34. Lincoln G. Reproductive seasonality and maturation throughout the complete life-cycle in the mouflon ram (*ovis musimon*). Anim Reprod Sci 1998;53:87-105.
35. Rhind S, McMillen S, Duff E, Hirst D, Wright S. Seasonality of meal patterns and hormonal correlates in red deer. Physiol Behav 1998;65:295-302.
36. Herndon J, Bein M, Nordmeyer D, Turner J. Seasonal testicular function in male rhesus monkeys. Horm Behav 1996;30:266-71.
37. Chan P, Hutz R, Dukelow W. Nonhuman primate in vitro fertilization: seasonality, cumulus cells, cyclic nucleotides, ribonucleic acid, and viability assays. Fertil Steril 1982;38:609-15.
38. Loscher W, Fiedler M. The role of technical, biological and pharmacological factors in the laboratory evaluation of anticonvulsant drugs. VI. Seasonal influences on maximal electroshock and pentylentetrazol seizure thresholds. Epilepsy Res 1996;25:3-10.
39. Sargeant J, Shoukri M, Martin S, Leslie K, Lissemore K. Investigating potential risk factors for seasonal variation: an example using graphical and spectral analysis methods based on the production of milk components in dairy cattle. Prev Vet Med 1998;36:167-78.
40. Yellon S, Fagoaga O, Nehlsen-Cannarella S. Influence of photoperiod on immune cell functions in the male Siberian hamster. Am J Physiol 1999;276:R97-102.
41. Demas G, Nelson R. Exogenous melatonin enhances cell-mediated, but not humoral, immune function in adult male deer mice (*Peromyscus maniculatus*). J Biol Rhythms 1998;13:245-52.
42. Feigin RD, San Joaquin VH, Haymond MW, Wyatt RG. Daily periodicity of susceptibility of mice to pneumococcal infection. Nature 1969;224:379-80.
43. Shackelford PG, Feigin RD. Periodicity of susceptibility to pneumococcal infection: influence of light and adrenocortical secretions. Science 1973;182:285-7.

44. Wongwiwat M, Sukapanit S, Triyanond C, Sawyer WD. Circadian rhythm of the resistance of mice to acute pneumococcal infection. Infect Immun 1972;5:442-8.
45. Sack R, Brandes R, Kendall A, Lewy A. Entrainment of free-running circadian rhythms by melatonin in blind people. N Engl J Med 2000;343:1070-7.
46. Sher L, Goldman D, Ozaki N, Rosenthal N. The role of genetic factors in the etiology of seasonal affective disorder. J Affect Disord 1999;53:203-10.
47. Eastman C, Young M, Fogg L, Liu L, Meaden P. Bright light treatment of winter depression: a placebo-controlled trial. Arch Gen Psychiatry 1998;55:883-9.
48. Pell J, Cobbe S. Seasonal variations in coronary heart disease. QJM 1999;92:689-96.
49. Ownby H, Frederick J, Mortensen R, Ownby D, Russo J. Seasonal variations in tumor size at diagnosis and immunological responses in human breast cancer. Invasion Metastasis 1986;6:246-56.
50. Calvo J, Rafil-El-Idrissi M, Pozo D, Guerrero J. Immunomodulatory role of melatonin: specific binding sites in human and rodent lymphoid cells. J Pineal Res 1995;18:119-26.
51. Boctor F, Charmy R, Cooper E. Seasonal differences in the rhythmicity of human male and female lymphocyte blastogenic responses. Immunol Invest 1989;18:775-84.
52. Maes M, Stevens W, Scharpe S, Bosmans E, De Meyer F, D'Hondt P, et al. Seasonal variation in peripheral blood leukocyte subsets and in serum interleukin-6, and soluble interleukin-2 and -6 receptor concentrations in normal volunteers. Experientia 1994;50:821-9.
53. Nelson R, Drazen D. Melatonin mediates seasonal adjustments in immune function. Reprod Nutr Dev 1999;39:383-98.
54. Paigen B, Ward E, Reilly A, Houten L, Gurtoo HL, Minowada J, et al. Seasonal variation of aryl hydrocarbon hydroxylase activity in human lymphocytes. Cancer Res 1981;41:2757-61.
55. Shadrin A, Marinich I, Taros L. Experimental and epidemiological estimation of seasonal and climato-geographical features of non-specific resistance of the organism to influenza. J Hyg Epidemiol Microbiol Immunol 1977;21:155-61.
56. Schulman J, Kilbourne E. Experimental transmission of influenza virus infection in mice. II. Some factors affecting the incidence of transmitted infections. J Exp Med 1963;118:267-75.
57. Swartz T, Skalska P, Gerichter C. Routine administration of oral polio vaccine in a subtropical area. Factors possibly influencing sero-conversion rates. J Hyg Camb 1972;70:719-26.
58. World Health Organization Collaborative Study Group on Oral Poliovirus V. Factors affecting the immunogenicity of oral poliovirus vaccine: a prospective evaluation in Brazil and the Gambia. J Infect Dis 1995;171:1097-106.
59. Deming M, Linkins R, Jaitch K, Hull H. The clinical efficacy of trivalent oral polio vaccine in the Gambia by season of vaccine administration. J Infect Dis 1997;175 s1:s254-7.
60. Schonberger L, McGowan JJ, Gregg M. Vaccine-associated poliomyelitis in the United States, 1961-1972. Am J Epidemiol 1976;104:202-11.
